# Paraneoplastic NMDA encephalitis, a case report and an extensive review of available literature

**DOI:** 10.1016/j.radcr.2023.11.087

**Published:** 2024-01-15

**Authors:** Hamza Alzghoul, Ferdous Kadri, Mohamed F. Ismail, Robeer Youssef, Mustafa Shamaileh, Ahmad R. Al-Assi, Liliya Adzhieva, Bashar Alzghoul

**Affiliations:** a University of Central Florida College of Medicine, Graduate Medical Education, Orlando, FL, USA; b Division of Pulmonary and Critical Care Medicine, Cedars Sinai Medical Center, Los Angeles, CA, USA; c Jordan University of Science and Technology College of Medicine, Irbid, Jordan; d Sixth of October University College of Medicine, Giza, Egypt; e The University of Jordan College of Medicine, Amman, Jordan; f Sechenov University, Moscow, Russia; g Division of Pulmonary, Critical Care and Sleep Medicine, University of Florida, Gainesville, FL, USA

**Keywords:** NMDAr encephalitis, Paraneoplastic, Renal cell carcinoma, RCC, Radiology

## Abstract

Anti-N-methyl-D-aspartate receptor (NMDAr) encephalitis is a prevalent autoimmune condition marked by diverse neuropsychiatric symptoms, primarily impacting young females. The exact mechanisms underlying the development of NMDAr encephalitis have not been fully elucidated. Nonetheless, studies have demonstrated that auto-antibodies targeting the NR1-NR2 subunits of the NMDAr can trigger receptor dysfunction within the central nervous system, thus giving rise to the associated symptoms. Notably, an association exists between NMDAr encephalitis and an underlying neoplastic condition, with approximately 38% of cases exhibiting this paraneoplastic relationship with ovarian teratomas being the most commonly associated malignancy.

While the association between NMDAr encephalitis and renal cell carcinoma (RCC) is exceedingly rare. This case report presents the clinical scenario of a 20-year-old female patient diagnosed with NMDAr encephalitis in conjunction with RCC discovered incidentally on a CT abdomen and pelvis performed to rule out an ovarian teratoma. The presented case underscores the importance of adopting a multidisciplinary approach in the diagnosis and treatment of NMDAr encephalitis, particularly when it is linked to an underlying malignancy. Furthermore, it emphasizes the significance of expanding our understanding of the molecular pathogenesis of NMDAr encephalitis to enhance patient care and optimize clinical outcomes. Additionally, a comprehensive review of the existing literature is included, summarizing all reported malignancies associated with NMDAr encephalitis.

## Introduction

Anti-N-methyl-D-aspartate receptor (NMDAr) encephalitis is a rare autoimmune condition identified by a broad spectrum of neuropsychiatric symptoms, predominantly affecting young females [1]. However, its clinical diagnosis remains insufficiently recognized, contributing to the complexity of managing this condition [1]. The clinical presentation of NMDAr encephalitis encompasses psychiatric symptoms, including agitation and hyperkinetic movements, as well as autonomic dysfunction and seizures [2].

The exact mechanisms underlying the development of NMDAr encephalitis have not been fully elucidated. Nonetheless, studies have demonstrated that auto-antibodies targeting the NR1-NR2 subunits of the NMDAr can induce receptor hypofunction or downregulation within the central nervous system, thus giving rise to the associated symptoms [3]. Notably, an association exists between NMDAr encephalitis and an underlying neoplastic condition, with approximately 38% of cases exhibiting this paraneoplastic relationship [4].

While the association between NMDAr encephalitis and renal cell carcinoma (RCC) is exceedingly rare [5], [6], [7]. This case report presents the clinical scenario of a 20-year-old female patient diagnosed with NMDAr encephalitis in conjunction with RCC. The presented case underscores the importance of adopting a multidisciplinary approach in the diagnosis and treatment of NMDAr encephalitis, particularly when it is linked to an underlying malignancy. Furthermore, it emphasizes the significance of expanding our understanding of the molecular pathogenesis of NMDAr encephalitis to enhance patient care and optimize clinical outcomes. Additionally, a comprehensive review of the existing literature is included, providing insights into potential malignancies associated with or causing paraneoplastic anti-NMDA receptor encephalitis, elucidating the underlying physiological mechanisms, discussing therapeutic strategies, and shedding light on the prognosis for individuals afflicted by this complex disorder.

## Case presentation

A 20-year-old African American female came to the emergency department (ED) with several symptoms that had been bothering her for several months. She had experienced paresthesia, insomnia, hyperkinetic muscle movements, and behavioral changes. She had visited other hospitals in the past month seeking help with her symptoms but had not found any relief. To manage her symptoms, she was given antiepileptic drugs and benzodiazepines, but her symptoms did not improve, and she started self-medicating with alcohol. When she came to the ED, she was exhibiting severe rhythmic movements in her extremities, a shortened attention span, and body rocking. She reported no use of illicit drugs. About a month ago, she was still able to do all her daily living activities.

Upon examination, her blood pressure was recorded as 121/67 mm Hg, heart rate as 122 beats per minute, and respiratory rate as 24 breaths per minute. She was noted to be febrile with an oral temperature of 38.5°C. The patient's body mass index was 38.16, indicative of morbid obesity.

Upon observation, the patient was visibly distressed, exhibiting bilaterally flailing arms and legs, with repeated muscle spasms causing alternate flexion and extension of her extremities. Due to her increasing psychomotor agitation, a comprehensive neurological examination was not performed, however, the rest of the exam was unremarkable except for muscle tenderness noted throughout the patient's body. The patient was later transferred to the medical intensive care unit (ICU). Despite the administration of sedative medications, including risperidone, midazolam, diazepam, and dexmedetomidine, her symptoms persisted and worsened over the course of her admission, leading to heavy sedation and intubation for airway protection and escalating agitation.

A comprehensive evaluation of the patient's worsening mental status and choreiform kinetic movements was initiated in the emergency department and continued in the medical ICU. Upon laboratory analysis, normocytic anemia with hyperchloremia, hyperkalemia, transaminitis, and metabolic acidosis were detected. The creatine kinase level was elevated at 40,100 mIU/L, which along with the results of urinalysis were consistent with rhabdomyolysis. Further testing, including serum osmolality, thyroid and iron studies, serum ammonia levels, and toxicology screening for methanol, ethanol, salicylates, and acetaminophen, all returned normal results. Additionally, negative results were obtained from syphilis and HIV screens. Blood and urine cultures did not indicate the presence of an infectious process. Brain computed tomography and electroencephalogram results were also inconclusive in identifying the underlying cause of the patient's symptoms.

**Fig. 1:**
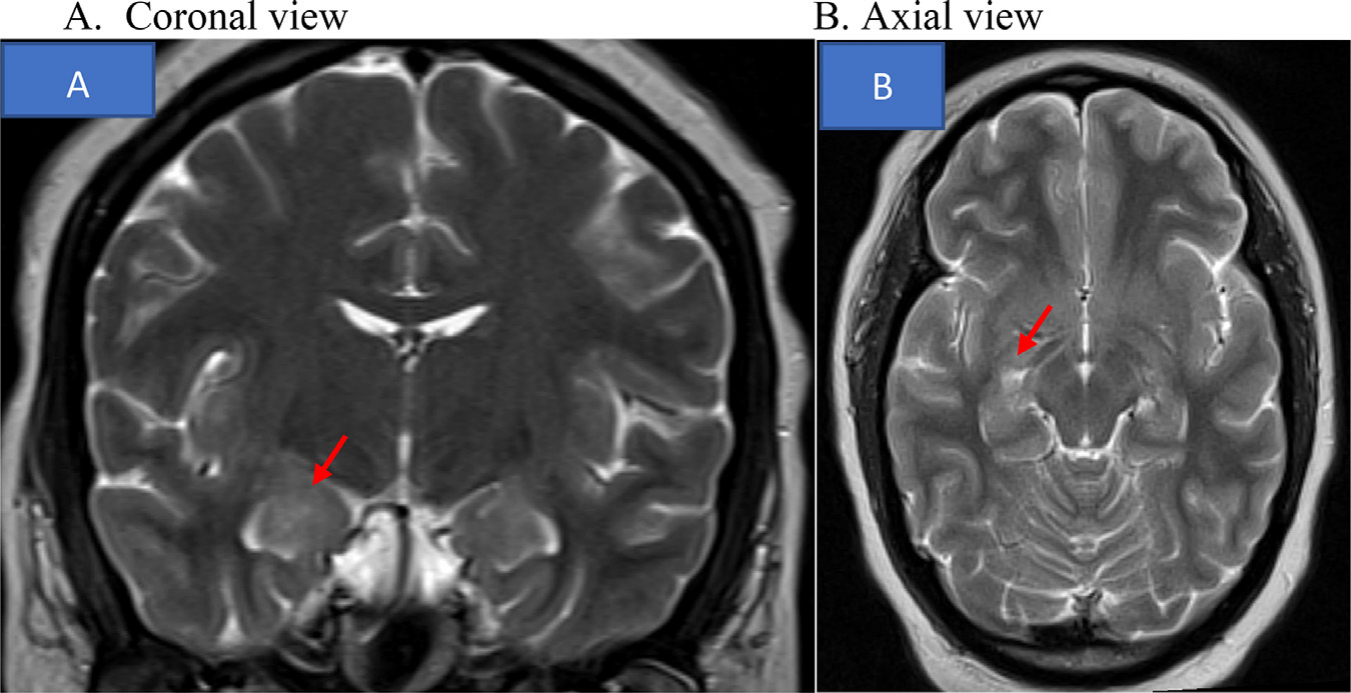
T2 Brain magnetic resonance imaging showing (A) Coronal view demonstrating increased intensity in the bilateral amygdala. (B) Hippocampal and corticospinal tracts hyperintensity.

Brain magnetic resonance imaging revealed increased T2 and FLAIR hyperintensity in the bilateral amygdala and corticospinal tracts (Fig. 1). To identify the cause of her worsening condition, a lumbar puncture with a thorough analysis of her cerebrospinal fluid (CSF) was conducted, including testing for arboviral and anti-N-methyl-D-aspartate (NMDA) receptor titers. The neurology team recommended testing for paraneoplastic autoantibodies using the Mayo Clinic paraneoplastic Antibody Panel (Mayo Medical Laboratories Test ID: PAVAL) as well. The patient required sedation, paralysis medication, and mechanical ventilation to manage her choreiform movements and muscle breakdown. During medical ICU admission, the test of NMDAr in serum and CSF was positive (1:640 Serum; 1:320 CSF), which raised concern for anti-NMDA receptor encephalitis of an unknown etiology.

**Fig. 2:**
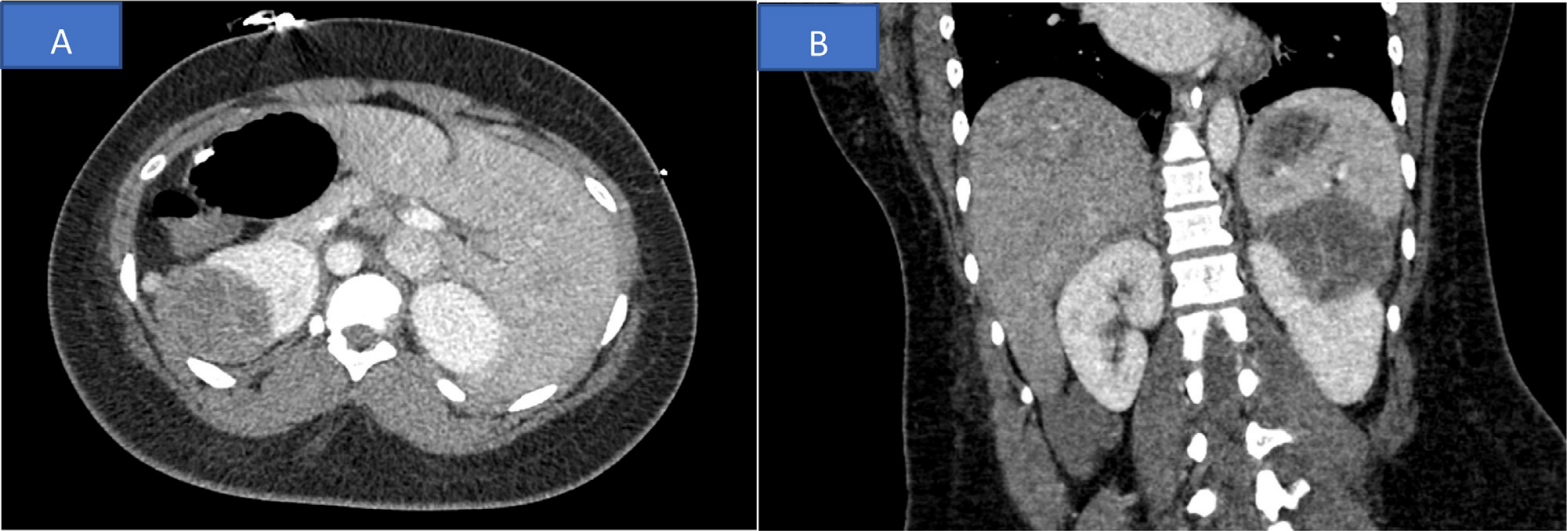
CT scan of the abdomen and pelvis showed a complex left renal mass (6.1 × 5.6 × 5.4 cm) with mixed densities consistent with a probable renal cell carcinoma. (A) Axial view, (B) Coronal view.

In light of previous studies linking NMDA receptor encephalitis to ovarian teratomas, a screening CT scan of the abdomen and pelvis, as well as a transvaginal ultrasound, were performed. However, no evidence of ovarian pathology was identified by the CT scan or ultrasound examination. Instead, a left renal mass (6.1 × 5.6 × 5.4 cm) consistent with a probable renal cell carcinoma was detected (Fig. 2). After 4 days of presentation, the patient was transferred to the neurological-ICU for further investigations and was maintained on deep sedation with propofol and fentanyl.

The patient was diagnosed with anti-NMDAr Encephalitis after excluding other explanations of her presentation. Despite improvement in her rhabdomyolysis through supportive care including adequate hydration, the patient continued to exhibit symptoms such as spasms, hyperkinetic movements, and an inability to protect her airway. During her 80-day admission in the neurological ICU, a multidisciplinary team-based approach was utilized with specialists from Neurology, Oncology, Urology, Hematology, and Surgical Services to guide her treatment. On hospital day 14, the patient also required a tracheostomy for prolonged mechanical ventilation. In addition, a gastrostomy tube was inserted on the same day. Additionally, the palliative team was consulted for further recommendations.

After thorough consideration and discussions with the patient's family, a total left nephrectomy was performed on the fifth hospital day (HD 5) and pathology confirmed the presence of renal cell carcinoma and incidental reno-medullary interstitial cell tumor. The patient was concurrently treated with 5 days of IV Solumedrol 1 g followed by 5 days of plasma exchange, however, due to the lack of improvement, neuroimmunology was consulted for potential immunotherapy. Subsequently, the patient was initiated on weekly rituximab 375 mg/m^2^ for 4 weeks starting from hospital day (HD) 20, which showed minor improvement leading to extubation.

Despite the use of various antipsychotic and anti-epileptic medications, the patient continued to experience dysautonomia, hyperkinetic movements, clonus, and agitation. On HD 54, a 4-day cyclophosphamide protocol with pulse dose steroids was initiated for the refractory NMDAr encephalitis and soon thereafter, the patient demonstrated improvement in her activity and reduced agitation. Her alertness continued to improve, allowing communication with family and healthcare providers. By HD 60, the patient showed decreased hyperkinetic movements and improved control over her extremities. IV sedation was eventually weaned off totally and her moderate agitation and refractory movements were managed with a combination of Valproic acid, Clonazepam, Pregabalin, clonidine, propranolol, and olanzapine.

Her serum NMDAr antibody titers continued to improve, with a reading of 1:160. On HD 69, the patient was able to follow 3-step commands and had full control over her extremities with 4/5 strength bilaterally. Given the positive response to the cyclophosphamide treatment, a protracted course of 10 doses was recommended by neuroimmunology. The patient was in the hospital receiving supportive care due to ongoing agitation during stressful periods. Despite this, she continued to make progress and recover her daily functions, such as communicating effectively, self-feeding, controlling her limbs, urinating, and transferring, among others. In order to mitigate symptoms, the patient practiced alternative meditation techniques under the guidance of the nursing staff. On occasion, she required the administration of intravenous benzodiazepines and minimal respiratory support through her tracheostomy collar. On the 74th hospital day, it was observed that the patient was able to sit up, wear her glasses, and watch the TV. When questioned about her memory, she appeared disoriented and reported having no recollection of the previous days of her hospital stay. The hospital staff provided reorientation each morning to help the patient better understand her diagnosis and current state.

Finally, a joint evaluation between the neuroimmunology and oncology teams was performed to determine the next course of action for the patient, given her persistent anti-NMDAr titers at 1:120. It was suggested that a positron emission tomography-computed tomography (PET-CT) scan be conducted in an outpatient setting to assess for residual renal cell carcinoma (RCC). If there was no further improvement observed in the patient's condition, the team also discussed the possibility of performing a bilateral oophorectomy as a supplementary management step.

## Discussion

### Causes of paraneoplastic NMRAr encephalitis

The etiology of anti-NMDAR encephalitis encompasses viral, tumor-related, and idiopathic origins, each capable of triggering the production of autoantibodies against NMDA receptors. The presence of plasma cells, IgG deposits, and reduced NMDAR levels observed in brain biopsies and autopsies, as well as the detection of clonal plasma cells producing anti-NMDAR in the cerebrospinal fluid (CSF), provide evidence for this autoimmune response [8]. Notably, NMDAr encephalitis often manifests as a paraneoplastic syndrome, with ovarian teratoma representing the most commonly associated tumor type.

A multi-institutional observational study conducted by Titulaer et al. [4] encompassed 220 patients with tumor-associated anti-NMDAR encephalitis, wherein 94% of the tumors were ovarian teratomas, 2% were extra ovarian teratomas, and the remaining 4% comprised 2 cases each of lung cancer, breast cancer, testicular tumors, ovarian carcinoma, thymic carcinoma, and pancreatic cancer [4].

Furthermore, a retrospective observational study by Bost et al. [9] documented 60 cases of tumor-associated anti-NMDAR receptor encephalitis, with ovarian teratomas accounting for 85% of the cases. The remaining patients (15%) exhibited other malignant tumors, including 3 cases of small cell lung carcinoma, 1 uterine adenocarcinoma, 1 prostate adenocarcinoma, 1 Hodgkin lymphoma, 1 pineal dysgerminoma, 1 neuroblastoma, and 1 pancreatic neuroendocrine tumor.

Interestingly, anti-NMDAR receptor encephalitis has also been associated with benign and precancerous lesions. Yin et al. [10] reported 3 cases of anti-NMDA receptor encephalitis in patients without underlying tumors but with prominent melanocytic nevi. The subsequent resection of these nevi was linked to patient recovery, suggesting that melanocytes in nevi may serve as potential autoantigens, eliciting an autoimmune response leading to encephalitis. Consequently, nevi resection in these patients resulted in improved response to immunotherapy and recovery. The authors highlighted the necessity for further clinical and experimental investigations to validate the treatment approach of resecting melanocytic nevi in anti-NMDAR encephalitis patients without concurrent tumors.

In contrast, paradoxically, anti-NMDAR encephalitis has also been associated with the resection of melanocytic nevi. Yang et al. [11] reported 2 cases of anti-NMDA encephalitis following the excision of melanocytic nevi. Both patients were females in their 20s, and initial screening tests for ovarian teratoma or other tumors yielded negative results. One patient received treatment with steroids and mycophenolate mofetil, while the other patient underwent first-line immunotherapy, and both eventually achieved recovery. The authors suggested that exposure to certain antigens on nevus cells resulting from nevus excision might activate an autoimmune response leading to the development of anti-NMDAR encephalitis. They emphasized the need for further research to evaluate the validity of this hypothesis.

This review primarily focuses on paraneoplastic anti-NMDA encephalitis. A comprehensive literature search was conducted to identify the types of tumors associated with anti-NMDAR encephalitis, and a summary can be found in Table 1.

**Table 1:** Tumors associated with NMDAr encephalitis.

Type of tumor and reference	Sex, age (years)	First brain MRI	Antibodies in CSF	Anti-NMDA receptor antibodies in *Serum*	Tumor discovery after or before symptoms onset and the time between the 2 events (if available)	Lines of management	Immunofluorescence of tumor cells for NMDA receptors	Improvement related to the tumor, outcome (Follow up duration)
Neuroblastoma Bost et al. [9]	M, 3	Hyperintensity on cerebellum T1, T2, and moderate hydrocephaly	Yes	NA	After, 2 mo	Surgery Chemotherapy, plasmapheresis	NA	mRS=5 at 9 mo no tumor response Death 10 mo after onset due to tumor progression
Pineal dysgerminoma Bost et al. [9]	M,9	Hydrocephaly, heterogenous pineal gland tumor	Yes	NA	Before, 9 mo	Surgery, Radiotherapy, Chemotherapy, IVIG, Rituximab, Cyclophosphamide	Yes	mRS=0 at 24 mo still alive at 30 mo
Prostate Adenocarcinoma Bost et al. [9]	M, 64	Normal	Yes	NA	After, 1.5 mo	Hormonotherapy IVIG Methylprednisolone Rituximab	Yes	mRS=4 at 6 mo Death at 6.5 mo due to meningitis
Pancreas neuroendocrine tumor Bost et al. [9]	F,50	Normal	Yes	Yes	After, 4 mo	Methylprednisolone IVIG Rituximab Surgery	Yes	mRS=0 at 24 mo Alive at 30 mo
Uterine Adenocarcinoma Bost et al. [9]	F,71	Normal	Yes	NA	Before, 5 mo	Surgery Chemotherapy IVIG Methylprednisolone Cyclophosphamide	NA	mRs=2 at 7 mo Tumor metastasis at 6 mo Death at 18 mo due to tumor progression
Hodgkin Bost et al. [9]	M,25	Normal	Yes	NA	After, 1.5 mo	IVIG Chemotherapy	No	Total remission mRS=4 at 6 mo Death at 8 mo due to unexplained reason
SCLC Bost et al. [9]	F, 67	Hyperintensity in both hippocampus on T2 FLAIR	Yes	NA	After, 8.8 mo	Methylprednisolone, Plasmapheresis Chemotherapy Radiotherapy Rituximab	No	mRS= 1 at 9 mo Tumor recurrence at 21 mo Death at 6 mo due to tumor progression
SCLC Bost et al. [9]	M,66	Hyperintensity in both hippocampus on T2 FLAIR	Yes	NA	After, 21 d	Methylprednisolone IVIG Plasmapheresis Chemotherapy	No	mRS = 5 at 1 mo Death at 1 mo due to tumor progression
SCLC Bost et al. [9]	F,62	Hyperintensity in both hippocampus on T2 FLAIR	Yes	NA	After, 25 d	Methylprednisolone IVIG Chemotherapy Rituximab	NA	mRS = 5 at 1 mo Death at 2 mo due to tumor progression
SCLC Coban et al. (2016) [12]	M, 62	Normal	Yes	Yes	After, 9 mo metastatic lesion in parotid gland), 15 mo the primary tumor SCLC	Steroids, IVIG Surgery for metastasis Therapy for primary cancer was NA	NA	Complete recovery at 4 mo following steroids and IVIG therapy. No symptoms after immunotherapy.
SCLC Jeraiby et al. (2016) [13]	F,62	Hyperintensity in temporal and occipital lobes extending to insula, cingulum, internal pallidum and subthalamic nuclei on FLAIR	Yes	Yes	After, NA	Corticosteroids, IVIG Rituximab Chemotherapy	Yes	No neurological improvement despite regression of the tumor. Death after 2 mo of hospitalization,3 mo after symptom onset, due to infections.
SCLC Kobayashi et al. (2020) [14]	M, 61	Hyperintensity in right insula on T2 and FLAIR	Yes	NA	After, NA	Methylprednisolone IVIG	Yes	Died 1 y later due to sepsis without consciousness recovery
SCLC Akanuma et al. (2022) [15]	F,72	Hyperintensity in basal ganglia and medial temporal lobes on FLAIR	Yes	NA	After, NA	Methylprednisolone	NA but qPCR was positive for GluN1 Gene expression in tumor tissue.	No improvement in methylprednisolone. Death after 23 d of admission due to cardiopulmonary arrest.
SCLC Uruha et al. (2012) [16]	M, 68	Normal	Yes	NA	After, NA	Methylprednisolone Chemotherapy was refused by the patient family.	NA	Could not be assessed since tumor was not treated. Death on day 188 due to respiratory failure.
Metastatic squamous cell carcinoma of unknown primary origin Coban et al. (2016) [12]	M, 64	Hyperintensity in both medial temporal lobes with no contrast enhancement	Yes	Yes	After, 5 mo left inguinal mass with unknown primary tumor.	Steroids IVIG Plasmapheresis Surgery Chemotherapy Radiotherapy	NA	5 y of follow-up, there was no neurological relapses, no inguinal mass recur, and no primary tumor identified on multiple PET scans.
Subependymoma Xiao et al. (2017) [17]	M,34	Heterogenous lesion in right lateral ventricle with Hypointensity on T1, and Hyperintensity on T2 and T2-Flair	Yes	No	After, NA	Conservative treatment Surgery (6 mo after symptoms onset)	Yes	Total recovery at 6 mo of conservative treatment and no relapse for 1 y after surgical resection.
Colon Adenocarcinoma Park et al. (2019) [18]	M,44	Hyperintense right mesial temporal and insula on T2 and Flair	Yes	Yes	After, NA	Steroids Surgery Chemotherapy	NA	His mentality status start improving as soon as the second day after tumor resection. Full recovery at 2 y.
Gastric Cancer Ding et al. (2017) [19]	M,55	NA	Yes	NA	After, 4 mo after admission but he had symptoms for 2 y	Methylprednisolone, IVIG prednisone Chemotherapy Surgery	NA	Disappearance of limb twitching which was the only residual finding after immunotherapy and chemotherapy, and before surgery. Total recovery at 2 mo of follow-up after surgery.
Carcinosarcoma with neuroendocrine differentiation of the uterus Hara et al. (2011) [20]	F,65	Hyperintense in bilateral limbic, mainly hippocampus and amygdala on FLAIR and diffusion imaging.	Yes	Yes	Tumor was discovered before symptoms and was thought to be Leiomyoma but resection 40 d after admission confirm Carcinosarcoma of the uterus.	Surgery	Yes	Death due to multiple organ failure.
Uterine large cell neuroendocrine carcinoma Kobayashi et al. (2017) [21]	F,44	Normal	Yes	NA	Before, 7 d but resection after 9 d .	Surgery Plasmapheresis, IVIG Chemotherapy	Yes	Improvement of consciousness 1 mo after treatment. Death at day 104 after admission due to multiple organ failure due to tumor recurrence and progression.
Hepatic neuroendocrine carcinoma Lim et al. (2017) [22]	M,65	Hyperintensity in left lentiform nucleus on T2	Yes	Yes	After, 1 mo	Methylprednisolone	NA	He had improvement following methylprednisolone therapy. Due to ST-elevation myocardial infarction, Peripheral vascular disease, and infected foot gangrene, resection and chemotherapy could not be performed. Death after 6 mo due to tumor progression.
Pheochromocytoma Park et al. (2020) [23]	F,78	Hyperintensity in both caudate nucleus on T2	Yes	NA	After, NA	Methylprednisolone, IVIG Surgery Rituximab	NA	Improvement after 10 d of tumor resection and huge further improvement after another 10 d. Follow up MRI 1 mo postoperatively show decrease in hyperintensity lesions.
Merkel cell Carcinoma Shalhout et al. (2020) [24]	M, 59	Normal	Yes	NA	Before, NA (Symptoms onset after resection but before radiotherapy)	Surgery IVIG, prednisone Rituximab Radiotherapy	NA	Recovery after surgery and immunotherapy. Latest MRI reveals neither abnormalities nor metastasis.
Chronic myelogenous leukemia Yu et al. (2022) [25]	M,23	CSF-containing arachnoid cyst in left temporal lobe	Yes	Yes	After, 1 year	Corticosteroids IVIG Tacrolimus Hydroxyurea	NA	Almost symptoms free 4 mo after initial treatment and 8 mo before tumor diagnosis.
Clear renal cell carcinoma Yang et al. [5]	M,54	Normal	Yes	Yes	After, 17 d	Methylprednisolone, IVIG Surgery Cyclophosphamide	NA	Almost full recovery 20 d after tumor resection. Relapse after 6 mo, treated with methylprednisolone, IVIG for 3 d, then oral prednisone for 8 mo. No relapse after 11 mo of no medications.
Hodgkin lymphoma Zandi et al. (2009) [26]	M, 49	Abnormal signal in both temporal lobes on T2 and FLAIR	Yes	Yes	Before, 7 y. The patient had one relapse 5 y before onset and another before 3 wk.	Radiotherapy Chemotherapy Steroids IVIG plasmapheresis	NA	His neurological function and MRI show improvement. He started the chemotherapy for his second relapse. 6 mo after his symptom's onset, his anterograde memory continues to improve.
Lung adenocarcinoma Wu et al. (2016) [27]	M, 60	Multiple acute infarctions in both cerebral and cerebellum lobes.	Yes	No	After, NA	Icotinib-hydrochloride Methylprednisolone	NA	Improvement started 3 d after Methylprednisolone. Negative anti-NMDA receptor antibodies in both serum and CSF at 7 mo of follow-up.
Ovarian mucinous cystadenoma Cho et al. (2019) [28]	F,23	Mild cortical edema	Yes	NO	After, NA (Thought to be teratoma at first)	Surgery IVIG, corticosteroids	NA	Discharge 9 d postoperatively and her neurological state continue improving in the following 2 mo.
Ovarian cystadenofibroma Sanmaneechai et al. (2013) [29]	F, 19	Normal	NA	Yes	After, NA	IVIG Plasmapheresis Surgery	NA	Huge improvement within 1 wk postoperatively Negative anti-NMDA receptor 10 d postoperatively. Back to her job 18 mo after discharge.
Melanocytic nevi Yin et al. [10]	F, 28	NA	Yes	No	After, NA	IVIG, Methylprednisolone Surgery Mycophenolate mofetil	Yes	The first episode was treated by immunotherapy and the symptoms relieved. Relapse after 8 mo, and antibodies were positive in CSF but not in serum. It was treated by tumor resection and Immunotherapy. Full recovery at 2 y of follow-up.
Melanocytic nevi Yin et al. [10]	M, Early 20s	NA	Yes	NA	After, NA	Surgery IVIG, Methylprednisolone Mycophenolate mofetil	Yes	Gradual recovery at 1 mo follow-up and stable neurological symptoms were stable at 1 y of follow-up.
Melanocytic nevi Yin et al. [10]	M, 22	NA	Yes	Yes	After, NA	Steroids Surgery IVIG, Methylprednisolone	No	The first episode was not screened for anti-NMDA receptor encephalitis and was treated with steroid, acyclovir, and antiepileptic, and he responded well. Relapse after 2 and half months and he was diagnosed with anti-NMDA receptor encephalitis. Antibodies were negative after 3 wk of surgery and full recovery at 1 y of follow-up.
Melanocytic nevi Yang et al. [11]	F, 25	Normal	Yes	Yes	Before, 5 wk	Surgery Methylprednisolone Mycophenolate mofetil	NA	She recovered gradually after immunotherapy and the follow-up is still ongoing.
Melanocytic nevi Yang et al. [11]	F, 20s	Normal	Yes	Yes	She had symptoms 5 mo before admission, but nevi resection was 2 wk before admission.	Surgery IVIG Methylprednisolone	NA	She gradually recovered after immunotherapy and antibodies in the CSF became negative but in the serum.
Primary CNS B cell lymphoma Yokota et al. (2022) [30]	F, 73	Hyperintensity in right temporal and bilateral frontal lobes on FLAIR	Yes	No	After, 8 wk	Surgery Radiotherapy	NO	The anti-NMDA receptor antibodies decreased after the tumor resection and radiotherapy. Her neurological symptoms show improvement.
Lymphomatosis cerebri Mariotto et al. (2019) [31]	M, 54	Normal	Yes	No	After, 24 mo on autopsy	Plasmapheresis, Cyclophosphamide	NA	Death at 24 mo.
Papillary thyroid carcinoma Mahadeen et al. (2019) [32]	F,29	Susceptibility artifact in the right parietal lobe presumed to be caused by a remote hemorrhage	NA	Yes	After, NA	Methylprednisolone, plasmapheresis Rituximab, IVIG Surgery	No	Improvement was noted following weeks of surgery. Continuous improvement at 8 mo of follow-up.
Papillary thyroid carcinoma Chakraborty et al. (2021) [33]	M, 36	Hyperintensity in bilateral temporal lobes, orbitofrontal lobes and claustra on T2	Yes	NA	After, NA	Methylprednisolone IVIG Rituximab Surgery	NA	The patient had huge improvement with mild residual symptoms post thyroidectomy.
Thyroid hyperplasia Yilmaz et al. (2014) [34]	F, 23	Hyperintensity in left parahippocampal and temporooccipital on T2-FLAIR	Yes	Yes	After, NA	Surgery Plasmapheresis	Yes	The patient had no relapses at 2 y of follow-up.
Thyroid hyperplasia Palleiko et al. (2022) [35]	F, 25	Hyperintensity in right cerebellum	Yes	NA	After, 6 mo	Methylprednisolone IVIG Surgery	NA	The patient was free of symptoms and had no relapses at 8 mo of follow-up.
Thymoma Inoue et al. (2018) [36]	F, 68	Hyperintensity in bilateral limbic areas on T2-FLAIR	Yes	NA	After, NA	Methylprednisolone Surgery	NA	Her symptoms start to improve 2 mo after the tumor resection and HDS-R score rose to 28/30. Huge Improvement at 20 mo of follow-up.

CNS, central nervous system; CSF, cerebrospinal fluid; F, female; FLAIR, fluid attenuated inversion recovery; HDS-R, Revised Hasegawa Dementia Scale; IVIG, intravenous immunoglobulin; M, male; MRI, magnetic resonance imaging; mRS, modified Rankin score; NA, not available; NMDA, N-methyl-D-aspartate; qPCR, quantitative polymerase chain reaction; SCLC, small cell lung carcinoma.

### Pathophysiology of paraneoplastic NMDAr encephalitis

NMDA receptors are heterotetrameric structures comprising essential GluN1 subunits and either GluN2 or GluN3 subunits in various combinations [37]. The GluN1 subunit has 8 isoforms, while the GluN2 and GluN3 subunits have 4 and 2 isoforms, respectively [[37], [38]]. In the context of anti-NMDAr encephalitis, autoantibodies target the extracellular domain of the GluN1 subunits [2]. The binding of these autoantibodies to the subunits results in a reversible reduction in surface expression of NMDA receptors, leading to receptor hypofunction [38]. This reduction occurs due to internalization of the NMDA receptor surface protein and selective capping mediated by the autoantibodies [39].

**Fig. 3:**
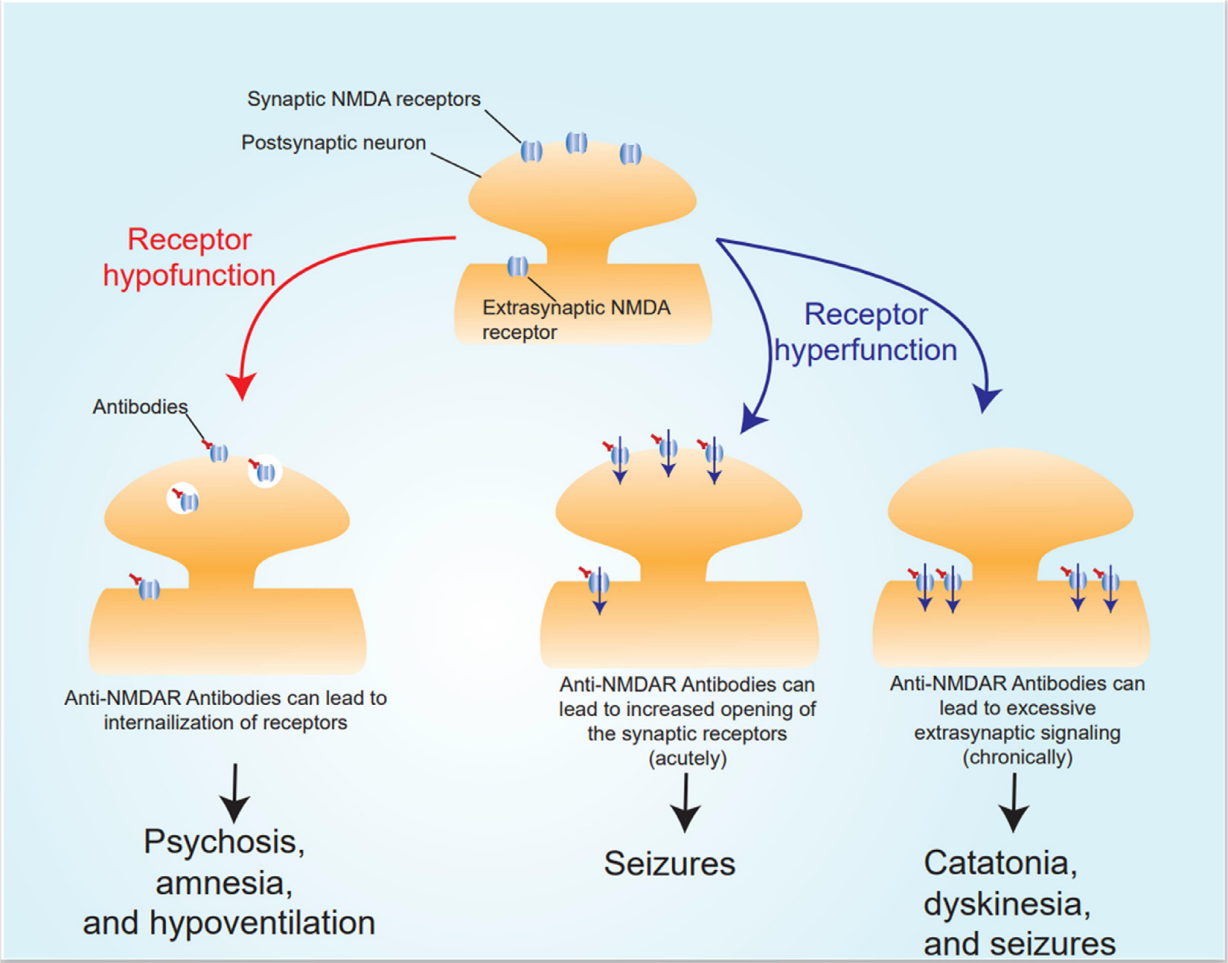
NMDAr encephalitis pathophysiology and related symptoms: Receptor hypofunction and receptor hypofunction.

However, this mechanism does not fully explain certain excitatory neurological symptoms observed in patients with NMDAr encephalitis, including seizures, dyskinesias, and catatonia. Published data suggests that NMDA receptor antibodies can initially prolong GluN1 channel opening, followed by a subsequent decrease in receptor surface expression. Moreover, the effects of the autoantibodies on the receptors can vary depending on the specific subtype, and at times, the anti-NMDAR antibodies may act as receptor agonists rather than antagonists, contributing to the aforementioned clinical symptoms [2]. Fig. 3 provides an illustrative representation of receptor hypofunction and receptor hyperfunction, as well as the associated symptoms in patients with NMDAr encephalitis.

In the context of RCC, we propose that anti-NMDAR encephalitis represents a rare paraneoplastic neurological syndrome associated with the tumor. Paraneoplastic neurological syndromes encompass a heterogeneous group of disorders unrelated to tumor metastasis, metabolic or nutritional deficits, coagulopathy, infections, or treatments [40]. NMDA receptors are expressed not only in the central nervous system but also in certain peripheral tissues, including the kidneys [[41], [42]]. The activation of these receptors in the renal tissue is believed to play a role in tubular and glomerular function [42]. In the case of RCC, we postulate that immune-related mechanisms may give rise to the production of autoantibodies against these receptors, leading to the manifestation of anti-NMDAR encephalitis. In the context of ovarian teratoma, it is well-documented that these tumors can contain mature and immature neuronal tissue expressing NMDA receptors. Thus, teratomas may contribute to the initiation of autoantibody synthesis, whereby these antibodies can subsequently cross-react with NMDA receptors present in the central nervous system [43].

### Management

The management of paraneoplastic anti-NMDA Receptor encephalitis is complex, time-sensitive, and often requires a multidisciplinary approach. It should target the tumor and NMDA antibody levels, and provide relief for the neuropsychiatric symptoms.

The optimal line of treatment consists of tumor removal and/or chemotherapy, combined with first-line immunotherapy (steroids, intravenous immunoglobulins, and plasmapheresis) [4]. Second-line immunotherapy (rituximab, cyclophosphamide, or both) improves outcomes for patients who did not respond to first-line immunotherapy and decreases the occurrence of relapses [[4], [44]]. The elimination of tumors, frequently coupled with immunotherapy, leads to a significant decrease in NMDAR antibody levels, with optimal outcomes observed when tumors are promptly removed [45].

Removing the tumor (usually an ovarian teratoma) is often an important step in the management of anti-NMDA receptor encephalitis [46]. An article describing a case of anti-NMDAr encephalitis in the setting of clear cell renal carcinoma reported that the patient markedly improved after undergoing nephron-sparing surgery for the tumor [47]. Another case of a 54-year-old patient with neuropsychiatric symptoms not responding to antiviral treatment for suspected viral encephalitis who had to be sedated due to worsening of his symptoms, was found to have a clear cell renal cell carcinoma. Following tumor resection (laparoscopic partial nephrectomy) and immunotherapy, the patient improved and was later discharged [5].

The presence of behavioral and psychotic symptoms can be a serious obstacle, as the condition is easily misdiagnosed [48]; which in turn delays the initiation of treatment. The administration of certain treatments such as plasma exchange is challenging in some patients with hyperkinesia or agitation, requiring them to be sedated. There is often a duration of a few weeks from symptom onset to laboratory confirmation of the diagnosis [49]. During this time, clinicians are left to prescribe symptomatic treatments, such as providing relief for seizures, catatonia, or perceptual disturbances. The aforementioned points re-emphasize the need for a multidisciplinary approach in managing patients with autoimmune encephalitis.

### Prognosis

Patient prognoses hinge on various factors, with those experiencing milder symptoms—defined as not necessitating ICU admission [[4], [25]]—and undergoing prompt initiation of immunotherapy and tumor removal [[4], [50]], being of younger age [51], and having lower antibody levels [[52], [53]] generally having more favorable outcomes.

Approximately 75% of individuals with anti-NMDA receptor encephalitis experience recovery or encounter mild consequences, while the remaining 25% may exhibit severe deficits or face eventual mortality [1]. Initial immunotherapy yields improvement in 53% of patients within the first 4 weeks, with 97% showing favorable outcomes at the 24-month mark. Among the 47% who do not respond to first-line treatment, those undergoing second-line immunotherapy (ie, rituximab, cyclophosphamide, or both) demonstrate better results compared to those persisting with initial treatment or receiving no additional immunotherapy [4].

A European study revealed that initiating treatment within 40 days of symptoms onset is associated with better outcomes [53]. Individuals with delayed treatment for 3 months or more suffered permanent hippocampal damage [54].

Elevated antibody titers are linked to adverse outcomes [55]. Changes in CSF titers are more closely tied to relapses than alterations in serum titers [55]. Several potential predictive biomarkers for anti-NMDAr encephalitis have been recognized, such as C-X-C motif chemokine 13 (CXCL13), cell-free mitochondrial DNA, interleukin-17, YKL-40 (Chitinase 3-like 1), Neuron-specific enolase (NSE), and S100 calcium-binding protein B (S100B). Elevated concentrations of these biomarkers in the CSF are associated with a higher likelihood of displaying inadequate responses to immunotherapy [[12], [56], [57], [58], [59]].

Despite the severity of symptoms, paraneoplastic anti-NMDAr encephalitis demonstrates a more favorable prognosis compared to most other paraneoplastic encephalitides [60].

## Conclusion

In conclusion, we present an exceedingly rare case of anti-NMDAr encephalitis in the setting of an incidentally discovered renal cell carcinoma in a 20-year-old female. Additionally, we performed an extensive review of existing literature on the pathophysiology, associated malignancies, management, and prognosis of paraneoplastic anti-NMDAr encephalitis.

## Patient consent

Informed consent was obtained from the patient's surrogate decision-maker (Mother) for the publication of this case report, ensuring confidentiality and voluntary participation.

Written consent was not obtained at the time of acquiring patient information, the consent was obtained verbally. The patient's legal guardian is currently untraceable, but approval to publish has been given by The University of Florida ethics committee, and by local legislation given that verbal consent was obtained from the patient's legal guardian.
